# Rising Trends in Obesity and Heart Failure: Related Mortality in the United States, 1999-2024

**DOI:** 10.1016/j.jacadv.2025.102303

**Published:** 2025-10-30

**Authors:** Maryam Sajid, Shaheer Qureshi, Zahra Imran, Taimor Mohammed Khan, Dua Ali, Saad Ahmed Waqas, Raheel Ahmed, Gregg C. Fonarow

**Affiliations:** a Department of Medicine, Dow University of Health Sciences, Karachi, Pakistan; b National Heart and Lung Institute, Imperial College London, London, United Kingdom; c Division of Cardiology, Department of Medicine, Geffen School of Medicine at UCLA, Los Angeles, California, USA

**Keywords:** CDC WONDER, disparities, heart failure, mortality trends, obesity

## Abstract

**Background:**

Obesity is a major public health crisis in the United States and a key contributor to heart failure (HF). Both conditions independently increase mortality risk, yet national trends in obesity and HF-related deaths remain underexplored across demographic and geographic subgroups.

**Objectives:**

The purpose of this study was to evaluate long-term trends in obesity and HF-related mortality among U.S. adults from 1999 to 2024, with emphasis on disparities by sex, race/ethnicity, age, urbanization, and region.

**Methods:**

Age-adjusted mortality rates (AAMRs) per 100,000 adults aged ≥25 years were extracted from the Centers for Disease Control and Prevention Wide-Ranging Online Data for Epidemiologic Research database. Deaths with both obesity and HF listed as underlying or contributing causes were included. Temporal trends were assessed using joinpoint regression to calculate annual percent change (APC) and average APC with 95% CIs.

**Results:**

From 1999 to 2024, 161,870 obesity and HF-related deaths were identified. The AAMR increased from 1.2 (95% CI: 1.1-1.2) to 4.5 (95% CI: 4.4-4.6), with an average APC of 5.3% (95% CI: 4.9-5.7; *P* < 0.001). Mortality was higher in men than women (5.0 vs 4.0). Non-Hispanic Black adults had the highest AAMR (7.7), followed by White (4.6) and Hispanic (2.2). The Midwest had the greatest burden (4.8), and rural areas exceeded urban ones. Adults ≥65 years had the highest rate (13.2 in 2024), while those aged 25 to 44 years saw a threefold increase.

**Conclusions:**

Obesity and HF-related mortality have more than tripled, with persistent disparities underscoring the need for urgent, equity-focused public health strategies.

Obesity has become a global public health crisis, with U.S. prevalence rising to 41.9%—a nearly 10% increase in the past decade.^1^ In 2023, over one in 5 U.S. adults were classified as obese.^2^ This surge has heightened the risk of noncommunicable diseases, including diabetes and hypertension,^3^ while imposing an economic burden of $173 billion annually.^4^ Similarly, in the United States, heart failure (HF) impacts 6.5 million people, projected to reach 8 million by 2030, with an annual cost of $30.7 billion.^5^^,^^6^ Obesity is a key driver of HF, contributing through metabolic and inflammatory changes that promote hypertension, insulin resistance, and myocardial dysfunction.^7^

Furthermore, projections indicate that by 2030, 20% of the U.S. population will be aged 65 years and older—57% of whom will have HF—this population remains at the highest risk for HF mortality.^8^^,^^9^ Recent analyses, performed in the United States, have shown that deaths related to obesity and HF are increasing at an alarming rate.^10^ However, the contemporary burden and trends regarding obesity and HF-related mortality have not yet been investigated. Epidemiological data assessing the demographic and regional distribution of obesity and HF-related deaths remain crucial to identify patients at highest risk who may benefit from targeted interventions. Therefore, the aim of the present study is to assess current trends in obesity and HF-related deaths over the past 2 and a half decades and determine differences by sex, age, race, ethnicity, urbanization, and census region, using the data from U.S. CDC's (Centers for Disease Control and Prevention) WONDER (Wide-Ranging Online Data for Epidemiologic Research) data set.^11^

## Methodology

### Study setting and population

This study utilized data from the CDC WONDER database, spanning January 1, 1999, to December 31, 2024.^11^ Death certificates from the Multiple Cause-of-Death Public Use records were analyzed to identify cases involving both obesity and HF. We defined relevant deaths as those where both obesity and HF were recorded as a contributing or underlying cause of death. We identified obesity cases using the International Classification of Diseases–Tenth Revision (ICD-10) codes E66.x, while HF was classified with codes I11.0, I13.0, I13.2, and I50.x.^12^^,^^13^ Our study focused on deaths occurring among individuals aged 25 years and older. Institutional Review Board approval was not required, as the study utilized publicly available, deidentified data provided by a government source.

### Data abstraction

We extracted data on deaths associated with obesity and HF, including demographic information, population sizes, year of death, and geographic location. Additional data elements included urban-rural classifications, states, regional categories, and locations of death (medical facility, home, hospice, or nursing home/long-term care facility). The urban-rural classifications followed the National Center for Health Statistics Urban-Rural Classification Scheme, and geographic regions were categorized according to the U.S. Census Bureau divisions: Northeast, Midwest, South, and West.^14^ Race and ethnicity were recorded according to death certificate entries following the standards of the U.S. Office of Management and Budget. The racial groups included non-Hispanic (NH) White, NH Black, and Hispanic or Latino.^15^ The analysis was also stratified by age groups, with specific attention to obesity and HF. The age groups considered include young adults (25-44 years), middle-aged adults (45-64 years), and older adults (65 years and above).^16^ For the primary cause of death, we used the rankable cause of death feature in the CDC WONDER database, which provides mortality counts and rates for the 15 leading causes of death among individuals with these conditions.

### Statistical analysis

To assess national trends in obesity and HF-related mortality, we calculated both crude mortality rates and age-adjusted mortality rates (AAMRs) per 100,000 population from 1999 to 2024, stratified by year, sex, race, state, and region. AAMRs were calculated by standardizing deaths to the 2000 U.S. population.^17^ 95% CIs were reported for all rates. We analyzed temporal trends using the Joinpoint Regression Program (Version 5.2.0, National Cancer Institute), which identifies significant changes in trends by fitting log-linear regression models across time segments.^18^ To study the annual trends in obesity and HF-related AAMRs in adults, we assessed the average annual percentage change (AAPC) and their relative 95% CIs. 95% CIs were calculated using the Tiwari modified gamma method, which accounts for variability in age-standardized rates and provides more accurate coverage when death counts are small. In contrast, crude mortality rates were accompanied by 95% CIs derived from the Poisson distribution, reflecting uncertainty based solely on the observed number of deaths without age adjustment.^19^, ^20^, ^21^ A trend was defined as increasing or decreasing if the slope significantly differed from zero, with significance evaluated using two-tailed *t*-tests. Joinpoint regression was applied to the full 1999-2024 data set; however, AAPC analyses were stratified into 3 periods—prepandemic (1999-2019), during the pandemic (2020-2021), and postpandemic (2022-2024)—to avoid the confounding impact of COVID-19 and to better reflect underlying disease trends. This approach is consistent with Siddiqi et al, who similarly evaluated mortality trends by separating prepandemic, during pandemic, and postpandemic intervals.^22^ A sensitivity analysis was conducted, considering obesity as either the primary or contributing cause of death, while HF was restricted to the primary cause. A *P* value ≤0.05 was considered statistically significant.

## Results

**Figure 1:**
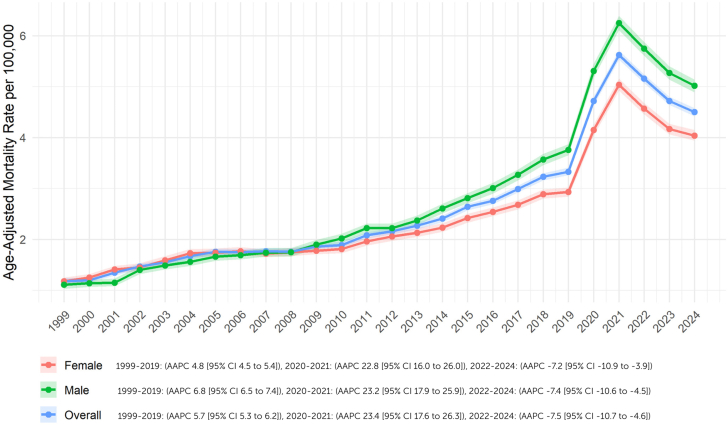
**Overall and Sex-Wise Trends** Trends and disparities in obesity and HF-related AAMR per 100,000 stratified by sex in the United States from 1999 to 2024. AAMR = age-adjusted mortality rate; HF = heart failure; AAPC = average annual percent change.

From 1999 to 2024, a total of 161,870 deaths were jointly attributed to both obesity and HF. Before the pandemic (1999-2019), the AAMR rose from 1.2 (95% CI: 1.1-1.2) in 1999 to 3.3 (95% CI: 3.3-3.4) in 2019, corresponding to an AAPC of 5.7% (95% CI: 5.3-6.2; *P* < 0.001). During the pandemic years (2020-2021), the AAMR sharply increased from 4.7 (95% CI: 4.6-4.8) in 2020 to 5.6 (95% CI: 5.5-5.7) in 2021, with an AAPC of 23.4% (95% CI: 17.6-26.3; *P* < 0.001). In the postpandemic period (2022-2024), the AAMR declined from 5.2 (95% CI: 5.1-5.2) in 2022 to 4.5 (95% CI: 4.4-4.6) in 2024, with an AAPC of −7.5% (95% CI: −10.7 to −4.6; *P* < 0.001) (Figure 1, Supplemental Tables 1 and 2). Sensitivity analysis—restricting HF to the primary cause of death while allowing obesity to be either a primary or contributing cause—demonstrated similar temporal patterns (Supplemental Figure 1).

### Sex

Men consistently exhibited higher AAMRs for obesity and HF-related mortality compared to women. In 1999, the AAMR for men was 1.1 (95% CI: 1.0-1.2), which increased to 3.8 in 2019 (95% CI: 3.7-3.9) (AAPC: 6.8%; 95% CI: 6.5-7.4; *P* < 0.001). Similarly, the AAMR for women rose from 1.2 (95% CI: 1.1-1.3) in 1999 to 2.9 (95% CI: 2.8-3.0) (95% CI: 2.8-3.0) in 2019 (AAPC: +4.8%; 95% CI: 4.5-5.4; *P* < 0.001). During the COVID-19 pandemic, AAMR in men rose from 5.3 (95% CI: 5.2-5.5) in 2020 to 6.3 (95% CI: 6.1-6.4) in 2021 (AAPC: +23.2%; 95% CI: 17.9-25.9; *P* < 0.001). In women, the AAMR increased from 4.2 (95% CI: 4.0-4.3) to 5.0 (95% CI: 4.9-5.2) (AAPC of +22.8%; 95% CI: 16.0-26.0; *P* < 0.001). Postpandemic, the AAMR declined in both sexes. Among men, it fell from 5.8 (95% CI: 5.6-5.9) in 2022 to 5.0 (95% CI: 4.9-5.1) in 2024 (AAPC: −7.4%; 95% CI: −10.6 to −4.5; *P* < 0.001), while in women it decreased from 4.6 (95% CI: 4.5-4.7) to 4.0 (95% CI: 3.9-4.2) (AAPC: −7.2%; 95% CI: −10.9 to −3.9; *P* < 0.001) (Figure 1, Supplemental Tables 2 and 10).

### Race

The AAMR for NH White individuals showed the most significant increase, rising from 1.1 (95% CI: 1.0-1.2) in 1999 to 3.4 (95% CI: 3.3-3.5) in 2019 (AAPC: +6.4%; 95% CI: 6.0-7.6; *P* < 0.001). Similarly, the Hispanic or Latino population experienced an increase from 0.7 (95% CI: 0.5-0.9) to 1.9 (95% CI: 1.8-2.1) (AAPC: +5.6%; 95% CI: 5.0-6.4; *P* < 0.001), as did the NH Black or African American population, with AAMRs rising from 2.3 (95% CI: 2.1-2.5) to 5.2 (95% CI: 4.9-5.4) (AAPC: +5.2%; 95% CI: 4.7-5.9; *P* < 0.001).

During the peak of the COVID-19 pandemic (2020-2021), all racial groups experienced sharp increases in AAMRs for obesity and HF. Among NH Black or African American individuals, the AAMR rose markedly from 8.2 (95% CI: 7.9-8.6) in 2020 to 9.9 (95% CI: 9.6-10.3) in 2021 (AAPC: +26.2%; 95% CI: 17.9-30.2; *P* = 0.002). Similarly, among NH White individuals, the AAMR increased from 4.6 (95% CI: 4.5-4.7) in 2020 to 5.6 (95% CI: 5.5-5.7) in 2021 (AAPC: +20.9%; 95% CI: 15.9-23.8; *P* < 0.001). Hispanic or Latino individuals also experienced a significant increase, with the AAMR rising from 3.1 (95% CI: 2.9-3.3) in 2020 to 3.4 (95% CI: 3.2-3.7) in 2021 (AAPC: +28.1%; 95% CI: 20.8-32.3; *P* < 0.001).

**Figure 2:**
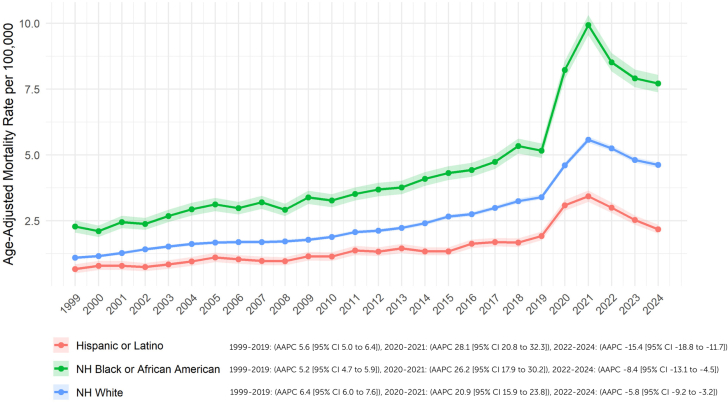
**Race-Wise Trends** Trends and disparities in obesity and HF-related AAMR per 100,000 stratified by race in the United States from 1999 to 2024. NH = non-Hispanic; other abbreviations as in Figure 1.

In the postpandemic period (2022-2024), all groups saw a reversal of this trend with consistent declines in AAMRs. For NH Black individuals, the AAMR declined from 8.5 (95% CI: 8.2-8.9) in 2022 to a provisional 7.7 (95% CI: 7.4-8.0) in 2024 (AAPC: −8.4%; 95% CI: −13.1 to −4.5; *P* = 0.001). Among NH White individuals, the AAMR dropped from 5.3 (95% CI: 5.2-5.4) in 2022 to 4.6 (95% CI: 4.5-4.7) in 2024 (AAPC: −5.8%; 95% CI: −9.2 to −3.2; *P* = 0.001). Hispanic or Latino individuals also showed a significant decrease, with the AAMR falling from 3.0 (95% CI: 2.8-3.2) in 2022 to 2.2 (95% CI: 2.0-2.4) in 2024 (AAPC: −15.4%; 95% CI: −18.8 to −11.7; *P* < 0. 0.001) (Figure 2, Supplemental Tables 3 and 10).

### Age-wise analysis

From 1999 to 2019, an increasing trend in mortality was observed across all age groups. Younger adults (25-44 years) experienced a notable increase in mortality, with AAMRs rising from 0.3 (95% CI: 0.3-0.4) in 1999 to 0.7 (95% CI: 0.6-0.7) in 2019 (AAPC: +4.6%; 95% CI: 4.2-5.2; *P* < 0.001). Middle-aged adults (45-64 years) showed a slightly higher increase, from 1.3 (95% CI: 1.2-1.4) to 3.4 (95% CI: 3.3-3.5) (AAPC: +5.0%; 95% CI: 4.5-5.7; *P* < 0.001), while older adults (65+ years) experienced the most significant rise in mortality, with AAMRs increasing from 3.1 (95% CI: 2.9-3.3) to 9.5 (95% CI: 9.3-9.8) (AAPC: +6.3%; 95% CI: 6.0-6.8; *P* < 0.001) (Supplemental Figure 2, Supplemental Tables 4 and 10).

During the peak of the COVID-19 pandemic (2020-2021), all age groups experienced a marked rise in AAMRs. Among individuals aged 25 to 44 years, the AAMR increased from 1.1 (95% CI: 1.0-1.1) in 2020 to 1.2 (95% CI: 1.1-1.3) in 2021 (AAPC: +26.0%; 95% CI: 17.6-30.2; *P* < 0.001). In the 45-64 age group, AAMR rose from 4.9 (95% CI: 4.7-5.0) to 5.8 (95%CI: 5.6-5.9) (AAPC: +24.7%; 95% CI: 14.8-29.0; *P* = 0.006). Among those aged 65 years and older, the AAMR surged from 13.1 (95% CI: 12.8-13.4) to 15.8 (95% CI: 15.5-16.1) (AAPC: +21.9%; 95% CI: 17.3-24.4; *P* < 0. 0.001).

In the postpandemic period (2022-2024), a consistent decline in AAMR was observed across all age groups. For 25–44-year-olds, the AAMR declined from 1.10 (95% CI: 1.0-1.2) in 2022 to 0.9 (95% CI: 0.8-1.0) in 2024 (AAPC: −10.0%; 95% CI: −14.6 to −6.0; *P* < 0.001). Among 45–64-year-olds, the rate dropped from 5.1 (95% CI: 4.9-5.2) to 4.4 (95% CI: 4.3-4.5) (AAPC: −8.8%; 95% CI: −14.7 to −5.1; *P* = 0.004). In the 65+ age group, the AAMR fell from 14.9 (95% CI: 14.6-15.2) to 13.2 (95% CI: 12.9-13.5) (AAPC: −6.1%; 95% CI: −8.7 to −3.5; *P* < 0. 0.001).

### Census region

From 1999 to 2019, mortality rates increased in all census regions. The Midwest region experienced the most significant rise, with the AAMR increasing from 1.3 (95% CI: 1.1-1.4) in 1999 to 3.8 (95% CI: 3.7-4.0) in 2019 (AAPC: +5.9%; 95% CI: 5.3-6.6; *P* < 0.001). Similarly, the South region saw a notable increase from 1.2 (95% CI: 1.1-1.2) in 1999 to 3.5 (95% CI: 3.4-3.7) in 2019 (AAPC: +5.9%; 95% CI: 5.6-6.5; *P* < 0.001), as did the West region from 1.4 (95% CI: 1.3-1.5) to 3.3 (95% CI: 3.2-3.5) (AAPC: +4.9%; 95% CI: 4.5-5.5; *P* < 0.001), and the Northeast region from 0.9 (95% CI: 0.8-1.0) to 2.4 (95% CI: 2.2-2.5) (AAPC: +5.6%; 95% CI: 5.2-6.1; *P* < 0.001) (Supplemental Figure 3, Supplemental Tables 5 and 10).

During the period from 2020 to 2021, all 4 Census Regions experienced a marked increase in AAMRs alongside AAPCs. In the Northeast, the AAMR rose from 3.4 (95% CI: 3.2-3.5) in 2020 to 3.6 (95% CI: 3.4-3.8) in 2021 (AAPC: +21.0%; 95% CI: 13.9-24.5; *P* = 0.003). The Midwest saw an increase from 5.2 (95% CI: 5.0-5.4) to 6.0 (95% CI: 5.7-6.2) (AAPC: +18.1%; 95% CI: 12.5-21.1; *P* = 0.005). The South exhibited the steepest rise, from 5.1 (95% CI: 5.0-5.2) to 6.4 (95% CI: 6.3-6.6) (AAPC: +27.2%; 95% CI: 21.1-30.7; *P* < 0.001). Similarly, the West experienced an increase from 4.7 (95% CI: 4.5-4.9) to 5.7 (95% CI: 5.5-5.9) (AAPC: +20.8%; 95% CI: 12.9-24.3; *P* = 0.004).

In contrast, the 2022 to 2024 period showed a reversal in trend across all regions. In the Northeast, the AAMR decreased from 3.5 (95% CI: 3.3-3.6) in 2022 to 3.1 (95% CI: 2.9-3.3) in 2024 (AAPC: −6.3%; 95% CI: −10.8 to −2.8; *P* = 0.004). The Midwest dropped from 5.5 (95% CI: 5.3-5.7) to 4.8 (95% CI: 4.6-5.0) (AAPC: −7.2%; 95% CI: −10.6 to −4.3; *P* < 0. 0.001). The South showed a similar decline from 5.8 (95% CI: 5.6-5.9) to 5.2 (95% CI: 5.0-5.3) (AAPC: −7.0%; 95% CI: −10.5 to −3.8; *P* < 0.001). The West also declined from 5.2 (95% CI: 5.0-5.4) to 4.3 (95% CI: 4.1-4.4) (AAPC: −8.5%; (95% CI: −13.0 to −4.9; *P* < 0. 0.001).

### Urbanization

From 1999 to 2019, nonmetropolitan areas had higher obesity and HF-related mortality than metropolitan areas, with overall AAMRs of 2.9 (95% CI: 2.9-3.0) and 1.9 (95% CI: 1.9-2.0), respectively. AAMRs in nonmetropolitan areas showed a greater increase compared to metropolitan areas from 1999 to 2019 (nonmetropolitan: AAPC: +6.1%; 95% CI: 5.7-6.7; *P* < 0.001; metropolitan: AAPC: +5.4%; 95% CI: 5.2-5.9; *P* < 0.001) (Supplemental Figure 4, Supplemental Table 6).

### State

**Figure 3:**
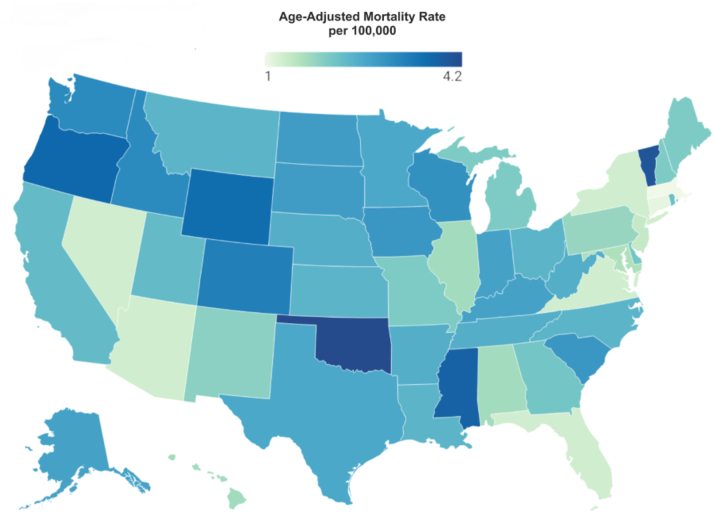
**States Map** Obesity and HF-associated AAMRs per 100,000 stratified by state in the United States from 1999 to 2019. Abbreviations as in Figure 1.

From 1999 to 2019, a significant difference in AAMRs for obesity and HF-related mortality was observed across different states, with the AAMRs ranging from 1.0 (95% CI: 0.9-1.0) in Massachusetts to 4.2 (95% CI: 4.0-4.4) in Oklahoma. States that fell into the top 90th percentile, such as Oklahoma, Vermont, Mississippi, Oregon, and Wyoming, had significantly higher AAMRs compared to states in the lower 10th percentile, including Florida, New York, Nevada, Connecticut, and Massachusetts (Figure 3, Supplemental Table 7).

### Place of death

Information on the location of death was available for 161,861 deaths from 1999 to 2024. Of these, 78,009 deaths occurred in medical facilities, and 53,781 deaths occurred in decedent’s home, 20,284 deaths occurred in nursing home/long-term care, 4,034 deaths (2.1%) occurred in hospices, 238 deaths occurred in unknown location and 5,515 deaths occurred in other locations (Supplemental Table 8).

### Underlying causes of death

We analyzed the top 15 leading underlying causes of death from 1999 to 2024 due to obesity and HF. Heart disease emerged as the most common cause, accounting for 73,275 deaths, followed by diabetes mellitus with 14,292 deaths and chronic lower respiratory disease with 13,521 deaths (Supplemental Table 9).

## Discussion

This study examined trends in obesity and HF-related mortality from 1999 to 2024, uncovering notable patterns and disparities. Over the 2 and a half decades, there has been an overall rise in mortality rates linked to obesity and HF, with a more pronounced among male populations compared to the general trend. Disparities were also observed across racial groups, with NH Black individuals showing the highest mortality rates. Geographically, higher mortality rates were consistently noted in the Midwest and rural areas, exceeding those in other regions. Furthermore, older adults (aged 65+ years) had the highest mortality and saw the greatest increase in AAMRs across all age groups (Table 1, Central Illustration).

**Table 1:** Frequency and Age-Adjusted Mortality Rates per 100,000 in Adults With Obesity and HF Concomitantly, Stratified by Sex, Age Group, Race, Census Region

Category	Deaths	Population	AAMR 1999 (95% CI)	AAMR 2024 (95% CI)	AAPC (95% CI)
Overall	161,870	4,247,219,476	1.2 (1.1-1.2)	4.5 (4.4-4.6)	5.3 (4.9-5.7)
Sex					
Men	81,577	2,044,831,030	1.1 (1.0-1.2)	5.0 (4.9-5.1)	6.2 (5.8-6.6)
Women	80,293	2,202,388,446	1.2 (1.1-1.3)	4.0 (3.9-4.1)	4.6 (4.2-5.1)
Age group					
Young adults (25-44 y)	12,217	1,763,170,919	0.3 (0.3-0.4)	0.9 (0.8-1.0)	4.3 (3.8-4.8)
Middle-aged adults (45-64 y)	59,068	1,611,231,257	1.3 (1.2-1.4)	4.4 (4.3-4.5)	4.7 (4.0-5.3)
Old adults (65+ y)	90,585	872,817,300	3.1 (2.9-3.3)	13.2 (12.9-13.5)	5.9 (5.6-6.3)
Race					
NH White	119,811	2,949,005,634	1.1 (1.0-1.2)	4.6 (4.5-4.7)	5.9 (5.5-6.9)
NH Black/African American	28,554	490,967,596	2.3 (2.1-2.5)	7.7 (7.4-8.0)	5.0 (4.4-5.5)
Hispanic/Latino	9,625	553,592,017	0.7 (0.5-0.9)	2.2 (2.0-2.4)	4.4 (3.9-5.1)
Census region					
Northeast	20,852	787,775,220	0.9 (0.8-1.0)	3.1 (2.9-3.3)	5.2 (4.8-5.6)
Midwest	38,116	922,845,632	1.3 (1.1-1.4)	4.8 (4.6-5.0)	5.1 (4.6-5.7)
South	64,353	1,565,644,413	1.2 (1.1-1.2)	5.2 (5.0-5.3)	5.8 (5.4-6.3)
West	38,549	970,954,211	1.4 (1.3-1.5)	4.3 (4.1-4.4)	4.4 (3.9-4.8)

AAMR = age-adjusted mortality rate; AAPC = average annual percent change; HF = heart failure; NH = non-Hispanic.

**Central Illustration:**
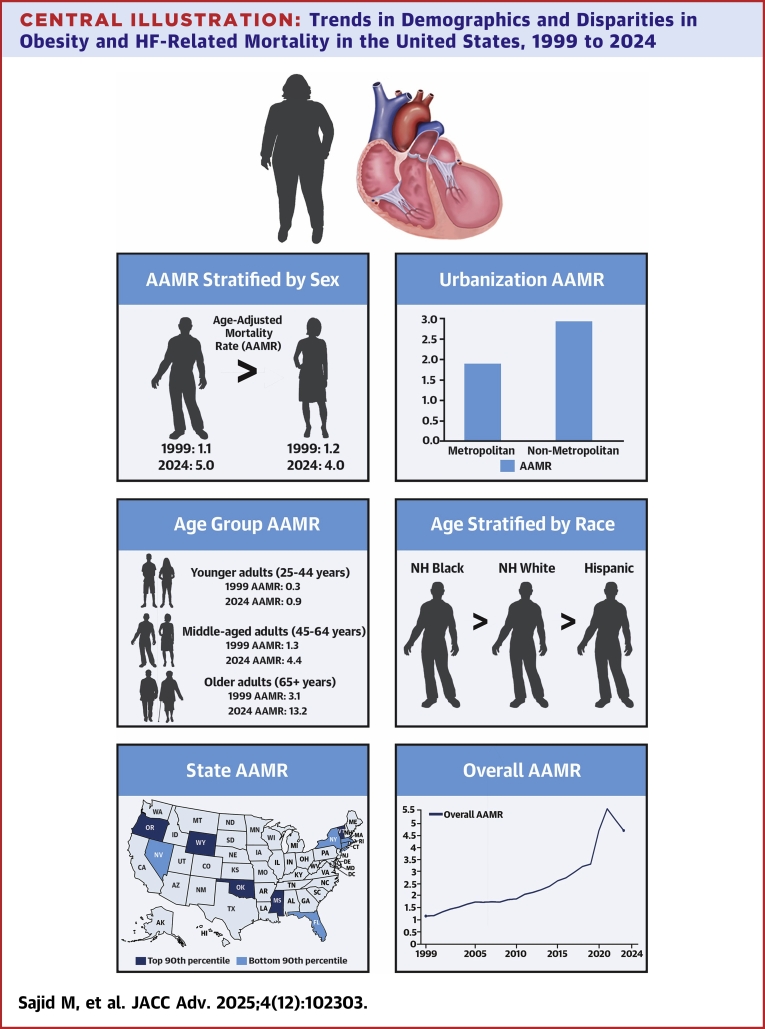
**Trends in Demographics and Disparities in Obesity and HF-Related Mortality in the United States, 1999 to 2024** Abbreviations as in Figures 1 and 2.

From 1999 to 2024, obesity and HF-related mortality followed a pattern of rapid increase, temporary slowdown, and then renewed acceleration. From 1999 to 2004 mortality rose rapidly, likely due to the increasing prevalence of obesity, poor dietary habits, and greater awareness and diagnosis of HF.^23^^,^^24^ Between 2004 and 2010, the rate of increase slowed, due to improved early and accurate diagnosis through advanced methods. However, from 2010 to 2019, mortality rates surged again, likely driven by the worsening obesity epidemic, an aging population, and the rise of metabolic disorders like diabetes and hypertension.^25^, ^26^, ^27^ The notable increase in AAMR across all demographic groups in 2020 can be linked to the COVID-19 pandemic. Patients with underlying health conditions, such as obesity and HF, faced a disproportionately higher risk of severe illness and mortality. Post-COVID-19, AAMR gradually declined but remained elevated compared to prepandemic levels.

Obesity contributes to HF through several mechanisms. These include cardiac workload, systemic inflammation, insulin resistance, and neurohormonal activation.^28^ These factors contribute to the progression of HF and increased mortality. In contrast, the “obesity paradox” suggests that mildly overweight or obese patients with HF may have better survival than those with normal weight.^29^ This paradox may be explained by greater metabolic reserves and higher lean body mass, which provide protective effects in the setting of HF.^30^

Our analysis identified that the AAMR associated with obesity and HF was marginally higher in men than in women. While obesity is a significant risk factor for HF in both men and women, the distribution of body fat differs between the sexes. Women tend to accumulate more subcutaneous fat, whereas men have greater intra-abdominal fat, largely due to hormonal influences.^31^ Intra-abdominal fat is considered the primary pathogenic fat depot clinically associated with CVD.^32^ Cardiac remodeling also varies: obese women often develop both concentric and eccentric left ventricular hypertrophy, while obese men more commonly show concentric hypertrophy alone—a pattern more strongly associated with cardiovascular mortality.^33^ Women generally have a reduced risk of cardiovascular disease, a difference that is largely attributed to the cardioprotective properties of estrogen.^34^ In men, poorer glycemic control, higher rates of diabetes, and harmful lifestyle behaviors such as smoking and heavy alcohol use—often reinforced by social and competitive pressures—may further heighten obesity- and HF-related mortality.^35^^,^^36^

Across racial groups, NH Black or African American individuals exhibited the highest mortality rates. Physiological differences partly explain this disparity. For instance, at the same body mass index (BMI), NH Black or African American individuals typically have less visceral fat and more lean mass than White individuals.^37^^,^^38^ These physiological variations underscore the importance of tailoring BMI thresholds to account for racial differences.^39^ Key societal contributors include disparities in access to stable and affordable housing, income inequality, and limited opportunities for quality education.^40^^,^^41^ Furthermore, these challenges are compounded by inequities in accessing affordable, nutritious food and safe environments for physical activity.^40^^,^^41^ Additionally, NH Black or African Americans, may also have a genetic predisposition to salt sensitivity, increasing their risk of high blood pressure and heart disease.^42^

In contrast to trends observed across all other demographic groups, we noted a considerable rise in AAMRs among younger and middle-aged adults throughout the study period. This rise is concerning, especially given the progress made in diagnostic and treatment methods over the last 2 decades such as microbiome profiling and strain imaging.^43^, ^44^, ^45^ Furthermore, lifestyle factors such as physical inactivity, chronic stress, and unhealthy eating habits have been found to heighten the risk obesity and HF at earlier ages.^18^^,^^36^ Research indicates that adolescents with obesity have significantly higher odds—7.15 times—of having a mental health disorder compared to their normal-weight peers, with a recent significant increase in mental health disorders observed among young adults.^46^, ^47^, ^48^ These findings underscore the importance of directing efforts toward young children in countries like U.S. grappling with an obesity epidemic, as interventions aimed at reducing BMI during childhood may prove more effective than those implemented in adulthood.^49^ Among older adults, obesity, in addition to contributing to HF, strains joints, increases fall risk, and leads to loss of independence, while also contributing to cognitive decline and dementia through vascular and metabolic damage.^50^^,^^51^

Our results show a significant disparity in obesity and HF-related mortality, with rural residents facing a higher burden than urban counterparts. This disparity is further exacerbated by factors such as lower income, limited education, restricted access to nutritious food, and fewer opportunities for physical activity, all of which contribute to higher obesity rates, especially in rural regions.^52^^,^^53^ Additionally, rural areas face greater challenges in HF prevention and treatment due to limited access to primary care providers and specialists, higher rates of uninsured residents, longer travel distances to health care facilities, and reduced internet connectivity, which hinders access to telehealth and health information.^52^ The COVID-19 pandemic further intensified these disparities, as rural areas a greater rise in AAMR compared to urban centers.

Effective management involves a combination of lifestyle modifications, pharmacological treatments, and, in some cases, surgical interventions. Guideline-directed medical therapy and the 2022 American Heart Association/American College of Cardiology guidelines for HF includes diuretics for fluid management and core medications such as angiotensin receptor-neprilysin inhibitors, beta-blockers, mineralocorticoid receptor antagonists, and sodium-glucose cotransporter 2 inhibitors, all of which help improve symptoms, prevent disease progression, and reduce hospitalizations and mortality.^54^^,^^55^ Individuals affected by obesity having a significantly higher risk of developing HF with preserved ejection fraction. Glucagon-like peptide-1 receptor agonists have shown promise in promoting weight loss and improving clinical outcomes in HF with preserved ejection fraction patients.^56^ American Heart Association and American College of Cardiology recommend structured lifestyle interventions to achieve at least 5% to 10% weight loss.^57^ Bariatric surgery is considered for individuals with severe obesity (BMI ≥40 kg/m^2^ or ≥35 kg/m^2^ with complications) who fail to achieve weight loss through nonsurgical methods.^57^ Beyond clinical strategies, coordinated public health measures are essential. These include policies that expand insurance coverage for obesity treatments, subsidies or vouchers to improve access to healthy foods in low-income communities, and investments to promote physical activity. Such measures address socioeconomic barriers and are essential to reduce the population-level burden of obesity and HF.

### Study Limitations

Firstly, the data relied solely on ICD-10 codes, which may be prone to inaccuracies or incomplete documentation. Misclassification or underreporting on death certificates is also possible and may have influenced our estimates. Furthermore, critical variables such as socioeconomic status, a significant determinant of health care outcomes, were not available for analysis. The CDC WONDER data set also did not include information on patients' baseline cardiovascular conditions or risk factors at the time of presentation, which restricts the depth of our conclusions. Additionally, the absence of data on the number of patients who received treatment for obesity and/or HF limited our ability to analyze the utilization of procedural and advanced therapies. Finally, we cannot discount the possibility that increased education and disease awareness may have improved the accuracy of obesity and HF diagnoses recorded on death certificates. The CDC WONDER database did not provide overall AAMR values for different racial groups; therefore, they were not included in the analysis. Urbanization data were only available till 2020; therefore, we extracted data up to 2019 to exclude the potential confounding impact of the COVID-19 pandemic. Similarly, state-level data were extracted only up to the year 2019. Lastly, this analysis includes provisional 2024 data, which are not final and may be subject to revision.

## Conclusions

Our study reveals an initial significant decline in obesity and HF-related mortality in the United States, followed by a recent resurgence, which was further exacerbated during the COVID-19 pandemic. Men and NH Black individuals were found to have the highest mortality risk, while rural areas and the Midwest region reported the highest AAMR compared to other parts of the country. Additionally, a concerning upward trend in mortality rates was observed among younger and middle-aged adults. These findings underscore the intricate relationship between cardiovascular conditions and social determinants of health. To improve outcomes across all demographic groups, health care strategies must prioritize health equity, particularly during public health crises such as the COVID-19 pandemic.

Perspectives **COMPETENCY IN MEDICAL KNOWLEDGE:** Obesity and HF-related mortality in the United States more than tripled from 1999 to 2024, with higher burdens in men, NH Black adults, rural areas, and the Midwest and South. Rising rates in younger adults highlight the need for earlier risk recognition and integrated weight management with guideline-directed HF therapy.

**TRANSLATIONAL OUTLOOK:** Reversing these trends will require dismantling systemic barriers to care and equity while strengthening prevention at both clinical and community levels. Future work should prioritize broad, sustainable strategies that address disparities and lessen the rising burden of obesity and HF-related mortality.

## Funding support and author disclosures

Dr Fonarow has done consulting for 10.13039/100000046Abbott, 10.13039/100002429Amgen, 10.13039/100004325AstraZeneca, 10.13039/100004326Bayer, 10.13039/100001003Boehringer Ingelheim, 10.13039/100014941Cytokinetics, Eli Lilly, 10.13039/100004331Johnson & Johnson, 10.13039/100004374Medtronic, 10.13039/100004334Merck, 10.13039/100004336Novartis, and 10.13039/100004319Pfizer. All other authors have reported that they have no relationships relevant to the contents of this paper to disclose.
