# Supplementary material for: Rising Trends in Obesity and Heart Failure: Related Mortality in the United States, 1999-2024
Source: JACC Adv. 2025 Oct 30;4(12):102303. doi: 10.1016/j.jacadv.2025.102303 (PMC12615744; doi:10.1016/j.jacadv.2025.102303)
Supplement: Supplemental Figures 1-6 and Tables 1-10 [file mmc1.docx]

**
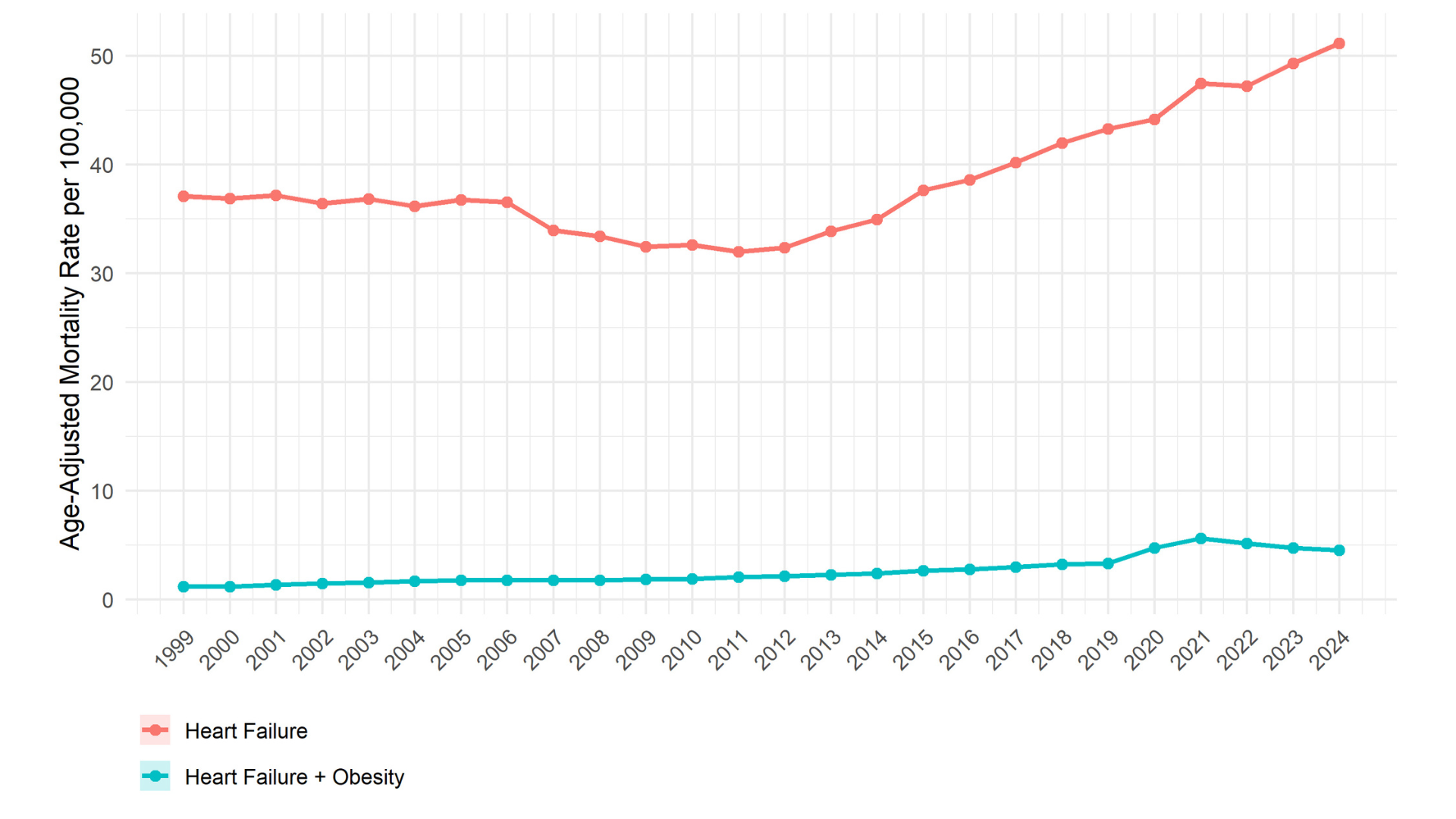
**

**Supplemental Figure 1.** Trends for heart failure as the underlying cause of death and HF-related mortality in patients with obesity as the Multiple Cause of Death in the United States from 1999 to 2024.

**
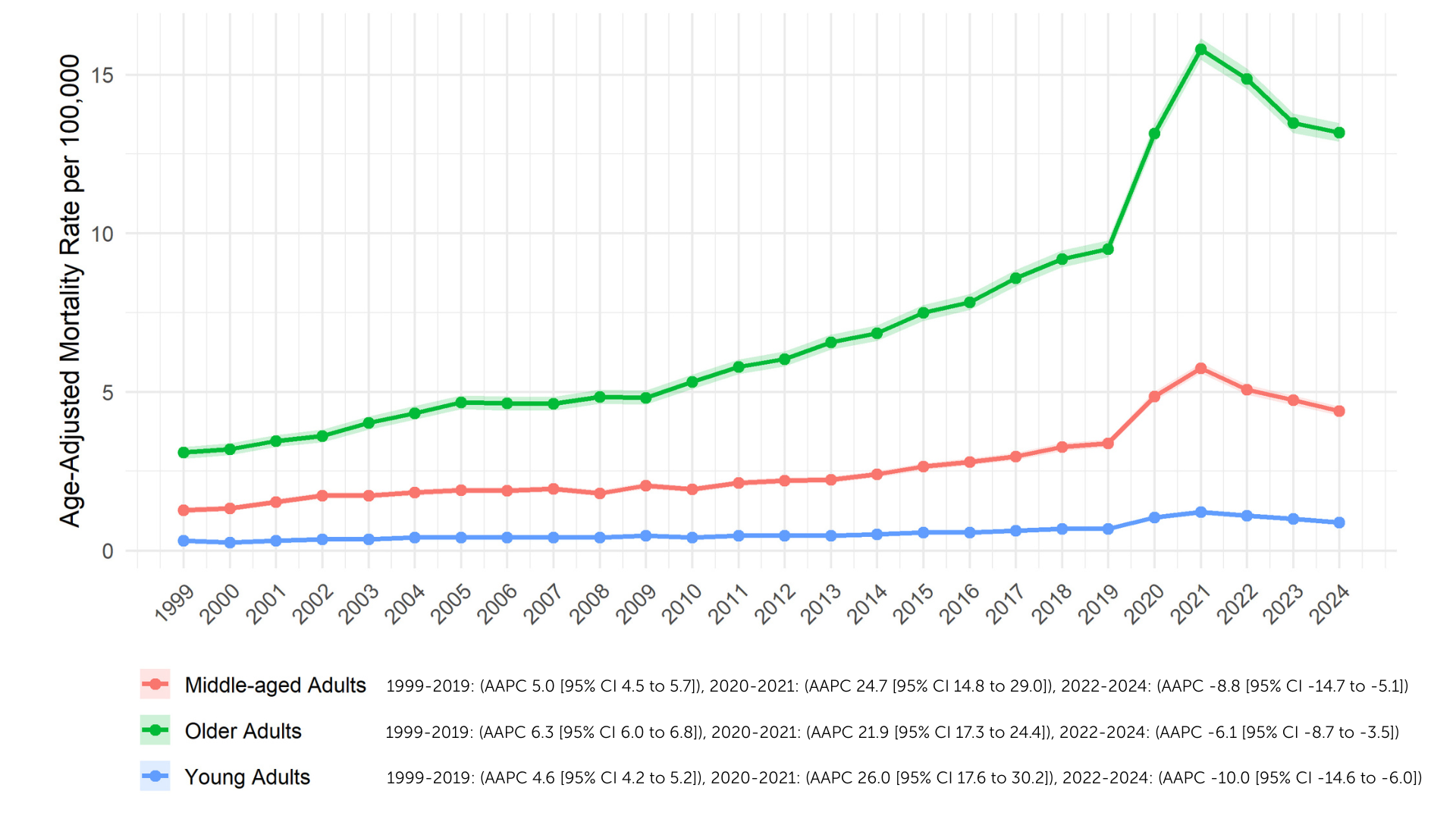
**

**Supplemental Figure 2.** Trends and disparities in obesity and heart failure-related AAMR per 100,000 in stratified by age in the United States from 1999 to 2024.


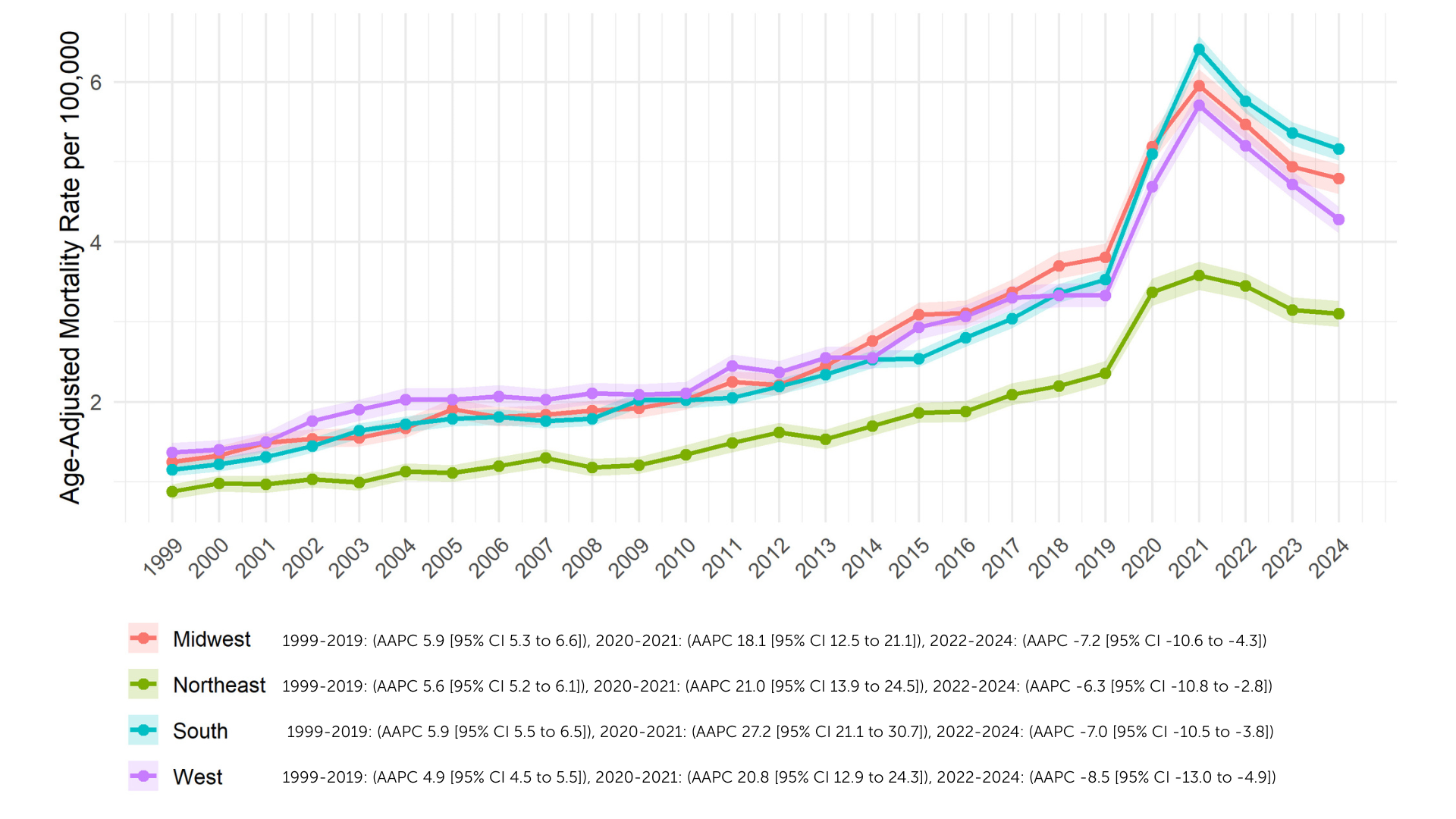


**Supplemental Figure 3.** Trends and disparities in obesity and heart failure-related AAMR per 100,000 in stratified by census region in the United States from 1999 to 2024.

**
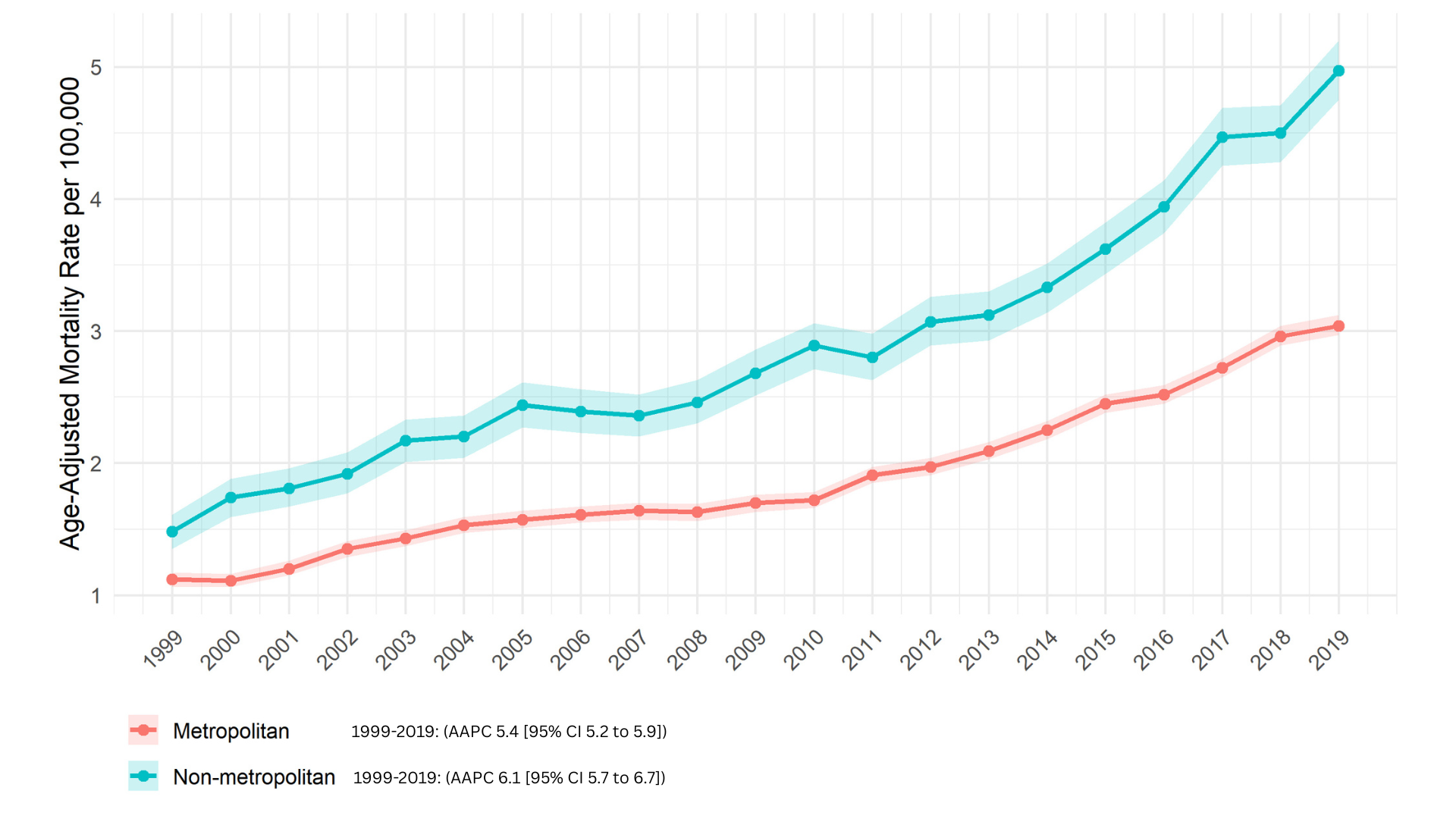
**

**Supplemental Figure 4.** Trends and disparities in obesity and heart failure-related AAMR per 100,000 in stratified by urbanization in the United States from 1999 to 2019.


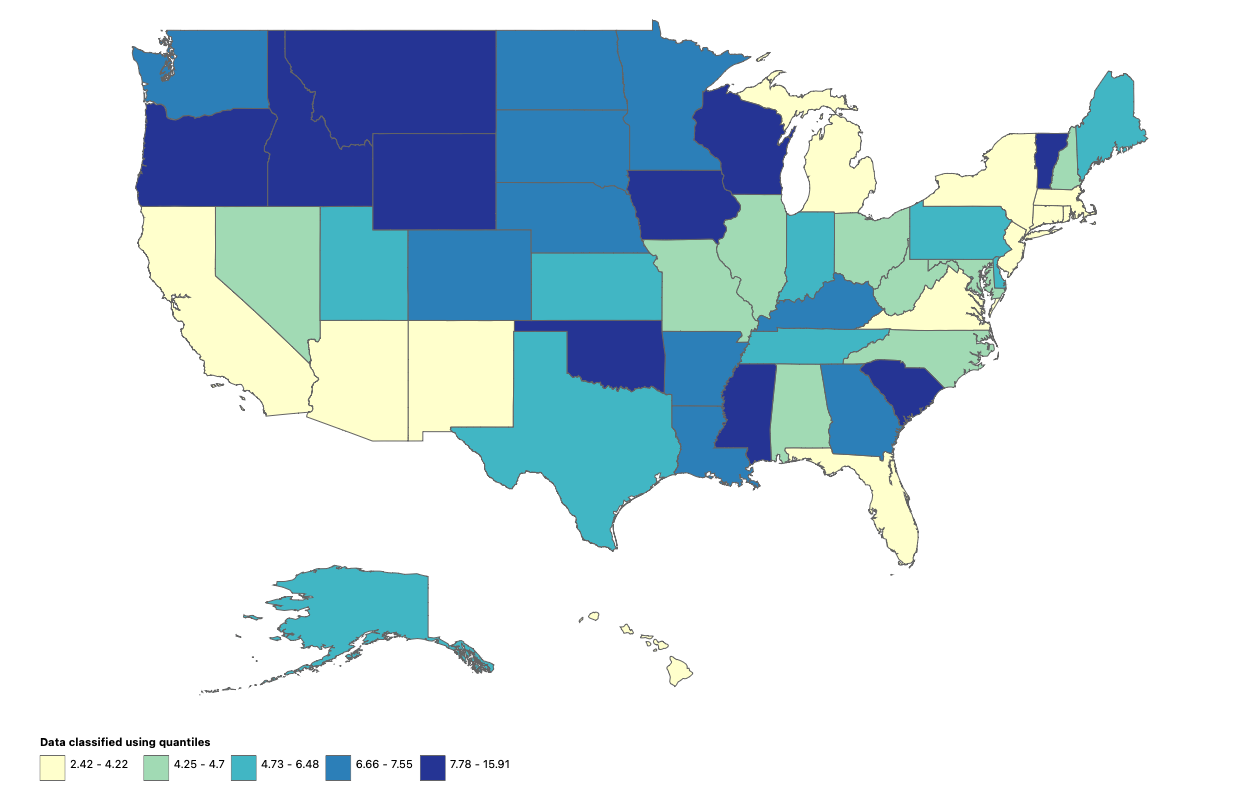


**Supplemental Figure 5**. Obesity and heart failure-associated AAMRs per 100,000 stratified by state in the United States from 2020 to 2021 (during Covid-19).


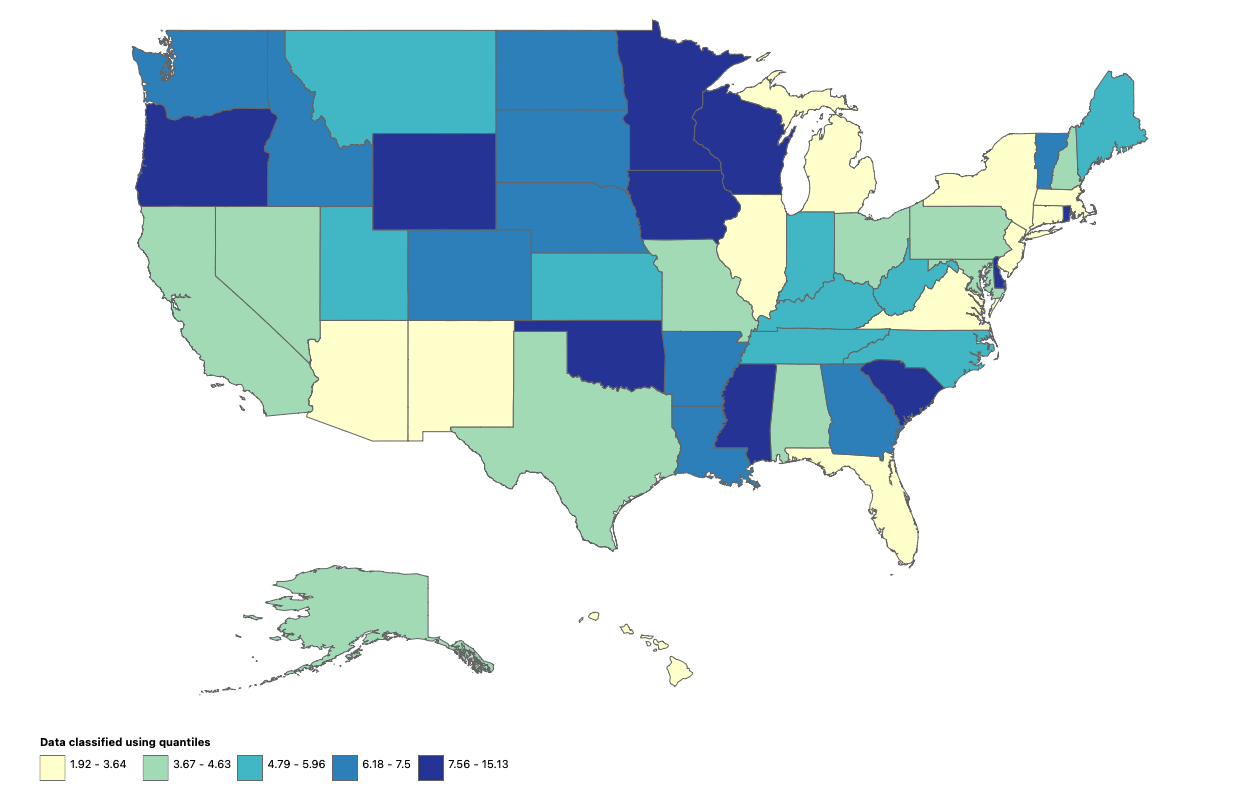


**Supplemental Figure 6**. Obesity and heart failure-associated AAMRs per 100,000 stratified by state in the United States from 2022 to 2024 (provisional) (post Covid-19).

| **Year** | **Overall** | **Female** | **Male** | **Hispanic or Latino** | **NH Black** | **NH White** | **Population** |
| --- | --- | --- | --- | --- | --- | --- | --- |
| 1999 | 2069 | 1179 | 890 | 78 | 387 | 1578 | 180408769 |
| 2000 | 2198 | 1270 | 928 | 96 | 370 | 1692 | 181984640 |
| 2001 | 2426 | 1423 | 1003 | 98 | 443 | 1855 | 184305128 |
| 2002 | 2712 | 1497 | 1215 | 106 | 457 | 2109 | 186208028 |
| 2003 | 2951 | 1664 | 1287 | 119 | 508 | 2270 | 188090429 |
| 2004 | 3234 | 1822 | 1412 | 139 | 583 | 2462 | 190205384 |
| 2005 | 3434 | 1913 | 1521 | 165 | 616 | 2590 | 192551384 |
| 2006 | 3519 | 1931 | 1588 | 166 | 613 | 2677 | 195019359 |
| 2007 | 3606 | 1949 | 1657 | 165 | 668 | 2715 | 197403777 |
| 2008 | 3676 | 2017 | 1659 | 167 | 623 | 2805 | 199795090 |
| 2009 | 3967 | 2062 | 1905 | 217 | 744 | 2924 | 202107016 |
| 2010 | 4142 | 2128 | 2014 | 218 | 728 | 3114 | 203891983 |
| 2011 | 4613 | 2355 | 2258 | 263 | 810 | 3462 | 206592936 |
| 2012 | 4828 | 2514 | 2314 | 274 | 859 | 3597 | 208826037 |
| 2013 | 5224 | 2691 | 2533 | 316 | 897 | 3896 | 211085314 |
| 2014 | 5705 | 2863 | 2842 | 317 | 1017 | 4252 | 213809280 |
| 2015 | 6325 | 3158 | 3167 | 326 | 1091 | 4782 | 216553817 |
| 2016 | 6821 | 3401 | 3420 | 411 | 1149 | 5108 | 218641417 |
| 2017 | 7476 | 3676 | 3800 | 458 | 1266 | 5581 | 221447331 |
| 2018 | 8167 | 4014 | 4153 | 463 | 1418 | 6101 | 223311190 |
| 2019 | 8672 | 4154 | 4518 | 554 | 1435 | 6505 | 224981167 |
| 2020 | 12366 | 5945 | 6421 | 938 | 2279 | 8783 | 226635013 |
| 2021 | 14814 | 7137 | 7677 | 1061 | 2745 | 10554 | 228238412 |
| 2022 | 13844 | 6669 | 7175 | 944 | 2381 | 10139 | 229508599 |
| 2023 | 12830 | 6202 | 6628 | 839 | 2267 | 9316 | 231529762 |
| 2024 (provisional) | 12251 | 5943 | 6308 | 727 | 2200 | 8944 | 231529762 |

**Supplemental Table 1.** Overall, sex stratified and race stratified obesity and heart failure-related mortality in the United States from 1999 to 2024.

| **Year** | **Overall** | **Female** | **Male** |
| --- | --- | --- | --- |
| 1999 | 1.2 (1.1-1.2) | 1.2 (1.1-1.3) | 1.1 (1.0-1.2) |
| 2000 | 1.2 (1.1-1.3) | 1.3 (1.2-1.3) | 1.1 (1.1-1.2) |
| 2001 | 1.4 (1.3-1.4) | 1.4 (1.3-1.5) | 1.1 (1.1-1.2) |
| 2002 | 1.5 (1.4-1.5) | 1.5 (1.4-1.5) | 1.4 (1.3-1.5) |
| 2003 | 1.6 (1.5-1.6) | 1.6 (1.5-1.7) | 1.5 (1.4-1.6) |
| 2004 | 1.7 (1.6-1.7) | 1.7 (1.6-1.8) | 1.6 (1.5-1.7) |
| 2005 | 1.8 (1.7-1.8) | 1.7 (1.7-1.8) | 1.6 (1.5-1.7) |
| 2006 | 1.8 (1.7-1.8) | 1.7 (1.7-1.8) | 1.7 (1.6-1.8) |
| 2007 | 1.8 (1.7-1.8) | 1.8 (1.7-1.8) | 1.7 (1.6-1.8) |
| 2008 | 1.8 (1.7-1.8) | 1.8 (1.7-1.8) | 1.8 (1.7-1.8) |
| 2009 | 1.9 (1.8-1.9) | 1.8 (1.7-1.9) | 1.9 (1.8-2.0) |
| 2010 | 1.9 (1.8-1.9) | 1.8 (1.7-1.9) | 2.0 (1.9-2.1) |
| 2011 | 2.1 (2.0-2.1) | 2.0 (1.9-2.0) | 2.2 (2.1-2.3) |
| 2012 | 2.2 (2.1-2.2) | 2.1 (2.0-2.1) | 2.2 (2.1-2.3) |
| 2013 | 2.3 (2.2-2.3) | 2.1 (2.1-2.2) | 2.4 (2.3-2.5) |
| 2014 | 2.4 (2.3-2.5) | 2.2 (2.1-2.3) | 2.6 (2.5-2.7) |
| 2015 | 2.6 (2.6-2.7) | 2.4 (2.3-2.5) | 2.8 (2.7-2.9) |
| 2016 | 2.8 (2.7-2.8) | 2.5 (2.5-2.6) | 3.0 (2.9-3.1) |
| 2017 | 3.0 (2.9-3.1) | 2.7 (2.6-2.8) | 3.3 (3.2-3.4) |
| 2018 | 3.2 (3.2-3.3) | 2.9 (2.8-3.0) | 3.6 (3.5-3.7) |
| 2019 | 3.3 (3.3-3.4) | 2.9 (2.8-3.0) | 3.8 (3.7-3.9) |
| 2020 | 4.7 (4.6–4.8) | 4.2 (4.0–4.3) | 5.3 (5.2–5.4) |
| 2021 | 5.6 (5.5–5.7) | 5.0 (4.9–5.2) | 6.2 (6.1–6.4) |
| 2022 | 5.2 (5.1–5.2) | 4.6 (4.5–4.7) | 5.8 (5.6–5.9) |
| 2023 | 4.7 (4.6–4.8) | 4.2 (4.1–4.3) | 5.3 (5.1–5.4) |
| 2024 (provisional) | 4.5 (4.4–4.6) | 4.0 (3.9–4.1) | 5.0 (4.9–5.1) |

**Supplemental Table 2.** Overall and sex-stratified obesity and heart failure-related AAMR per 100,000 in the United States from 1999 to 2024.

| **Year** | **Hispanic or Latino** | **NH Black or African American** | **NH White** |
| --- | --- | --- | --- |
| 1999 | 0.7 (0.5-0.9) | 2.3 (2.1-2.5) | 1.1 (1.0-1.2) |
| 2000 | 0.8 (0.6-1.0) | 2.1 (1.9-2.3) | 1.2 (1.1-1.2) |
| 2001 | 0.8 (0.6-1.0) | 2.5 (2.2-2.7) | 1.3 (1.2-1.3) |
| 2002 | 0.8 (0.6-0.9) | 2.4 (2.2-2.6) | 1.4 (1.4-1.5) |
| 2003 | 0.8 (0.7-1.0) | 2.7 (2.4-2.9) | 1.5 (1.5-1.6) |
| 2004 | 1.0 (0.8-1.1) | 2.9 (2.7-3.2) | 1.6 (1.6-1.7) |
| 2005 | 1.1 (0.9-1.3) | 3.1 (2.9-3.4) | 1.7 (1.6-1.7) |
| 2006 | 1.0 (0.9-1.2) | 3.0 (2.7-3.2) | 1.7 (1.6-1.8) |
| 2007 | 1.0 (0.8-1.1) | 3.2 (3.0-3.4) | 1.7 (1.6-1.8) |
| 2008 | 1.0 (0.8-1.1) | 2.9 (2.7-3.2) | 1.7 (1.7-1.8) |
| 2009 | 1.2 (1.0-1.3) | 3.4 (3.1-3.6) | 1.8 (1.7-1.9) |
| 2010 | 1.1 (1.0-1.3) | 3.3 (3.0-3.5) | 1.9 (1.8-2.0) |
| 2011 | 1.4 (1.2-1.6) | 3.5 (3.3-3.8) | 2.1 (2.0-2.1) |
| 2012 | 1.3 (1.2-1.5) | 3.7 (3.4-3.9) | 2.1 (2.1-2.2) |
| 2013 | 1.5 (1.3-1.6) | 3.8 (3.5-4.0) | 2.2 (2.2-2.3) |
| 2014 | 1.3 (1.2-1.5) | 4.1 (3.8-4.4) | 2.4 (2.3-2.5) |
| 2015 | 1.3 (1.2-1.5) | 4.3 (4.1-4.6) | 2.7 (2.6-2.7) |
| 2016 | 1.6 (1.5-1.8) | 4.4 (4.2-4.7) | 2.8 (2.7-2.8) |
| 2017 | 1.7 (1.5-1.9) | 4.7 (4.5-5.0) | 3.0 (2.9-3.1) |
| 2018 | 1.7 (1.5-1.8) | 5.3 (5.1-5.6) | 3.3 (3.2-3.3) |
| 2019 | 1.9 (1.8-2.1) | 5.2 (4.9-5.4) | 3.4 (3.3-3.5) |
| 2020 | 3.1 (2.9-3.3) | 8.2 (7.9-8.6) | 4.6 (4.5-4.7) |
| 2021 | 3.4 (3.2-3.6) | 9.9 (9.6-10.3) | 5.6 (5.5-5.7) |
| 2022 | 3.0 (2.8-3.2) | 8.5 (8.2-8.9) | 5.3 (5.2-5.4) |
| 2023 | 2.5 (2.4-2.7) | 7.9 (7.6-8.2) | 4.8 (4.7-4.9) |
| 2024 (provisional) | 2.2 (2.0-2.4) | 7.7 (7.4-8.0) | 4.6 (4.5-4.7) |

.

**Supplemental Table 3.** Obesity and heart failure-related AAMR per 100,000 stratified by race in the United States from 1999 to 2024.

| **Year** | **Young Adults (25-44)** | **Middle-Aged Adults (45-64)** | **Old Adults (65+)** |
| --- | --- | --- | --- |
| 1999 | 0.3 (0.3-0.4) | 1.3 (1.2-1.4) | 3.1 (2.9-3.3) |
| 2000 | 0.3 (0.2-0.3) | 1.3 (1.2-1.4) | 3.2 (3.0-3.4) |
| 2001 | 0.3 (0.3-0.4) | 1.5 (1.4-1.6) | 3.5 (3.3-3.6) |
| 2002 | 0.4 (0.3-0.4) | 1.7 (1.6-1.8) | 3.6 (3.4-3.8) |
| 2003 | 0.4 (0.3-0.4) | 1.7 (1.6-1.8) | 4.0 (3.8-4.2) |
| 2004 | 0.4 (0.4-0.5) | 1.8 (1.7-1.9) | 4.3 (4.1-4.6) |
| 2005 | 0.4 (0.4-0.5) | 1.9 (1.8-2.0) | 4.7 (4.5-4.9) |
| 2006 | 0.4 (0.4-0.5) | 1.9 (1.8-2.0) | 4.6 (4.4-4.9) |
| 2007 | 0.4 (0.4-0.5) | 2.0 (1.9-2.1) | 4.6 (4.4-4.9) |
| 2008 | 0.4 (0.4-0.5) | 1.8 (1.7-1.9) | 4.9 (4.6-5.1) |
| 2009 | 0.5 (0.4-0.5) | 2.1 (2.0-2.2) | 4.8 (4.6-5.0) |
| 2010 | 0.4 (0.4-0.5) | 1.9 (1.8-2.0) | 5.3 (5.1-5.5) |
| 2011 | 0.5 (0.4-0.5) | 2.1 (2.0-2.2) | 5.8 (5.6-6.0) |
| 2012 | 0.5 (0.4-0.5) | 2.2 (2.1-2.3) | 6.0 (5.8-6.3) |
| 2013 | 0.5 (0.4-0.5) | 2.2 (2.1-2.3) | 6.6 (6.3-6.8) |
| 2014 | 0.5 (0.5-0.6) | 2.4 (2.3-2.5) | 6.9 (6.6-7.1) |
| 2015 | 0.6 (0.5-0.6) | 2.7 (2.5-2.8) | 7.5 (7.2-7.7) |
| 2016 | 0.6 (0.5-0.6) | 2.8 (2.7-2.9) | 7.8 (7.6-8.1) |
| 2017 | 0.6 (0.6-0.7) | 3.0 (2.9-3.1) | 8.6 (8.3-8.9) |
| 2018 | 0.7 (0.6-0.7) | 3.3 (3.1-3.4) | 9.2 (8.9-9.5) |
| 2019 | 0.7 (0.6-0.7) | 3.4 (3.3-3.5) | 9.5 (9.3-9.8) |
| 2020 | 1.1 (1.0-1.1) | 4.9 (4.7-5.0) | 13.1 (12.8-13.4) |
| 2021 | 1.2 (1.1-1.3) | 5.8 (5.6-5.9) | 15.8 (15.5-16.1) |
| 2022 | 1.1 (1.0-1.2) | 5.1 (4.9-5.2) | 14.9 (14.6-15.2) |
| 2023 | 1.0 (0.9-1.1) | 4.7 (4.6-4.9) | 13.5 (13.2-13.8) |
| 2024 (provisional) | 0.9 (0.8-1.0) | 4.4 (4.3-4.5) | 13.2 (12.9-13.5) |

**Supplemental Table 4.** Obesity and heart failure-related AAMR per 100,000 stratified by age group in the United States from 1999-2024.

| **Year** | **Northeast** | **Midwest** | **South** | **West** |
| --- | --- | --- | --- | --- |
| 1999 | 0.9 (0.8-1.0) | 1.3 (1.1-1.4) | 1.2 (1.1-1.2) | 1.4 (1.3-1.5) |
| 2000 | 1.0 (0.9-1.1) | 1.3 (1.2-1.4) | 1.2 (1.1-1.3) | 1.4 (1.3-1.5) |
| 2001 | 1.0 (0.9-1.1) | 1.5 (1.4-1.6) | 1.3 (1.2-1.4) | 1.5 (1.4-1.6) |
| 2002 | 1.0 (0.9-1.1) | 1.5 (1.4-1.7) | 1.5 (1.4-1.6) | 1.8 (1.6-1.9) |
| 2003 | 1.0 (0.9-1.1) | 1.6 (1.4-1.7) | 1.6 (1.5-1.7) | 1.9 (1.8-2.0) |
| 2004 | 1.1 (1.0-1.2) | 1.7 (1.6-1.8) | 1.7 (1.6-1.8) | 2.0 (1.9-2.2) |
| 2005 | 1.1 (1.0-1.2) | 1.9 (1.8-2.0) | 1.8 (1.7-1.9) | 2.0 (1.9-2.2) |
| 2006 | 1.2 (1.1-1.3) | 1.8 (1.7-1.9) | 1.8 (1.7-1.9) | 2.1 (1.9-2.2) |
| 2007 | 1.3 (1.2-1.4) | 1.8 (1.7-2.0) | 1.8 (1.7-1.9) | 2.0 (1.9-2.2) |
| 2008 | 1.2 (1.1-1.3) | 1.9 (1.8-2.0) | 1.8 (1.7-1.9) | 2.1 (2.0-2.2) |
| 2009 | 1.2 (1.1-1.3) | 1.9 (1.8-2.1) | 2.0 (1.9-2.1) | 2.1 (2.0-2.2) |
| 2010 | 1.3 (1.2-1.5) | 2.0 (1.9-2.2) | 2.0 (1.9-2.1) | 2.1 (2.0-2.3) |
| 2011 | 1.5 (1.4-1.6) | 2.3 (2.1-2.4) | 2.1 (2.0-2.2) | 2.5 (2.3-2.6) |
| 2012 | 1.6 (1.5-1.7) | 2.2 (2.1-2.4) | 2.2 (2.1-2.3) | 2.4 (2.2-2.5) |
| 2013 | 1.5 (1.4-1.7) | 2.5 (2.3-2.6) | 2.3 (2.2-2.4) | 2.6 (2.4-2.7) |
| 2014 | 1.8 (1.6-1.8) | 2.8 (2.6-2.9) | 2.5 (2.4-2.6) | 2.6 (2.4-2.7) |
| 2015 | 1.9 (1.7-2.0) | 3.1 (2.9-3.2) | 2.5 (2.4-2.7) | 2.9 (2.8-3.1) |
| 2016 | 1.9 (1.8-2.0) | 3.1 (3.0-3.3) | 2.8 (2.7-2.9) | 3.1 (2.9-3.2) |
| 2017 | 2.1 (1.9-2.2) | 3.4 (3.2-3.5) | 3.0 (2.9-3.2) | 3.3 (3.1-3.5) |
| 2018 | 2.2 (2.1-2.3) | 3.7 (3.5-3.9) | 3.4 (3.2-3.5) | 3.3 (3.2-3.5) |
| 2019 | 2.4 (2.2-2.5) | 3.8 (3.7-4.0) | 3.5 (3.4-3.7) | 3.3 (3.2-3.5) |
| 2020 | 3.4 (3.2-3.5) | 5.2 (5.0-5.4) | 5.1 (5.0-5.2) | 4.7 (4.5-4.9) |
| 2021 | 3.6 (3.4-3.8) | 6.0 (5.7-6.2) | 6.4 (6.3-6.6) | 5.7 (5.5-5.9) |
| 2022 | 3.5 (3.3-3.6) | 5.5 (5.3-5.7) | 5.8 (5.6-5.9) | 5.2 (5.0-5.4) |
| 2023 | 3.2 (3.0-3.3) | 4.9 (4.8-5.1) | 5.4 (5.2-5.5) | 4.7 (4.5-4.9) |
| 2024 (provisional) | 3.1 (2.9-3.3) | 4.8 (4.6-5.0) | 5.2 (5.0-5.3) | 4.3 (4.1-4.4) |

**Supplemental Table 5.** Obesity and heart failure-related AAMR per 100,000 stratified by census region in the United States from 1999 to 2024.

| **Year** | **Metropolitan** | **Non-Metropolitan** |
| --- | --- | --- |
| 1999 | 1.1 (1.1-1.2) | 1.5 (1.4-1.6) |
| 2000 | 1.1 (1.1-1.2) | 1.7 (1.6-1.9) |
| 2001 | 1.2 (1.1-1.3) | 1.8 (1.7-2.0) |
| 2002 | 1.4 (1.3-1.4) | 1.9 (1.8-2.1) |
| 2003 | 1.4 (1.4-1.5) | 2.2 (2.0-2.3) |
| 2004 | 1.5 (1.5-1.6) | 2.2 (2.0-2.4) |
| 2005 | 1.6 (1.5-1.6) | 2.4 (2.3-2.6) |
| 2006 | 1.6 (1.6-1.7) | 2.4 (2.2-2.6) |
| 2007 | 1.6 (1.6-1.7) | 2.4 (2.2-2.5) |
| 2008 | 1.6 (1.6-1.7) | 2.5 (2.3-2.6) |
| 2009 | 1.7 (1.6-1.8) | 2.7 (2.5-2.9) |
| 2010 | 1.7 (1.7-1.8) | 2.9 (2.7-3.1) |
| 2011 | 1.9 (1.9-2.0) | 2.8 (2.6-3.0) |
| 2012 | 2.0 (1.9-2.0) | 3.1 (2.9-3.3) |
| 2013 | 2.1 (2.0-2.2) | 3.1 (2.9-3.3) |
| 2014 | 2.3 (2.2-2.3) | 3.3 (3.1-3.5) |
| 2015 | 2.5 (2.4-2.5) | 3.6 (3.4-3.8) |
| 2016 | 2.5 (2.4-2.6) | 3.9 (3.7-4.1) |
| 2017 | 2.7 (2.7-2.8) | 4.5 (4.3-4.7) |
| 2018 | 3.0 (2.9-3.1) | 4.5 (4.3-4.7) |
| 2019 | 3.0 (3.0-3.1) | 5.0 (4.8-5.2) |

**Supplemental Table 6.** Obesity and heart failure-related AAMR 100,000 stratified by urban-rural classification in the United States from 1999 to 2024.

| **State** | **Age Adjusted Rate (95% CI)** |
| --- | --- |
| Alabama | 1.7 (1.6-1.8) |
| Alaska | 2.7 (2.3-3.1) |
| Arizona | 1.3 (1.3-1.4) |
| Arkansas | 2.5 (2.4-2.7) |
| California | 2.3 (2.2-2.3) |
| Colorado | 3.3 (3.2-3.5) |
| Connecticut | 1.1 (1.0-1.2) |
| Delaware | 2.1 (1.8-2.3) |
| District of Columbia | 2.0 (1.7-2.4) |
| Florida | 1.3 (1.3-1.3) |
| Georgia | 2.1 (2.0-2.2) |
| Hawaii | 1.7 (1.5-1.9) |
| Idaho | 3.1 (2.8-3.3) |
| Illinois | 1.7 (1.6-1.7) |
| Indiana | 2.7 (2.6-2.8) |
| Iowa | 2.9 (2.7-3.0) |
| Kansas | 2.4 (2.2-2.6) |
| Kentucky | 2.7 (2.6-2.9) |
| Louisiana | 2.4 (2.3-2.5) |
| Maine | 2.0 (1.8-2.2) |
| Maryland | 1.6 (1.5-1.7) |
| Massachusetts | 1.0 (0.9-1.0) |
| Michigan | 2.0 (1.9-2.1) |
| Minnesota | 2.6 (2.5-2.7) |
| Mississippi | 3.8 (3.6-4.0) |
| Missouri | 2.0 (1.9-2.0) |
| Montana | 2.4 (2.2-2.7) |
| Nebraska | 2.5 (2.3-2.7) |
| Nevada | 1.3 (1.2-1.4) |
| New Hampshire | 2.0 (1.8-2.2) |
| New Jersey | 1.4 (1.3-1.4) |
| New Mexico | 1.9 (1.7-2.0) |
| New York | 1.3 (1.2-1.3) |
| North Carolina | 2.4 (2.3-2.5) |
| North Dakota | 2.8 (2.5-3.2) |
| Ohio | 2.4 (2.4-2.5) |
| Oklahoma | 4.2 (4.0-4.4) |
| Oregon | 3.7 (3.6-3.9) |
| Pennsylvania | 1.8 (1.8-1.9) |
| Rhode Island | 2.2 (1.9-2.4) |
| South Carolina | 2.9 (2.8-3.0) |
| South Dakota | 2.8 (2.5-3.1) |
| Tennessee | 2.5 (2.4-2.6) |
| Texas | 2.6 (2.5-2.6) |
| Utah | 2.3 (2.1-2.4) |
| Vermont | 4.0 (3.6-4.4) |
| Virginia | 1.3 (1.3-1.4) |
| Washington | 3.1 (3.0-3.2) |
| West Virginia | 2.5 (2.3-2.7) |
| Wisconsin | 3.0 (2.9-3.1) |
| Wyoming | 3.6 (3.2-4.0) |

**Supplemental Table 7.** Obesity and heart failure-related AAMR per 100,000 stratified by state in the United States from 1999 to 2019.

| **Place of Death** | **Deaths** | **% of Total Deaths** |
| --- | --- | --- |
| Medical Facility | 78,009 | 48.2% |
| Decedent's home | 53,781 | 33.2% |
| Hospice facility | 4,034 | 2.5% |
| Nursing home/long-term care | 20,284 | 12.5% |
| Other | 5,515 | 3.4% |
| Place of death unknown | 238 | 0.1% |
| Total | 161,861 | 100% |

**Supplemental Table 8.** Obesity and heart failure-related mortality among United States adults stratified by place of death from 1999 to 2024.

| **UCD - 15 Leading Causes of Death** | **Deaths** |
| --- | --- |
| Diseases of heart (I00-I09,I11,I13,I20-I51) | 73,275 |
| Diabetes mellitus (E10-E14) | 14,292 |
| Chronic lower respiratory diseases (J40-J47) | 13,521 |

**Supplemental Table 9**. Obesity and heart failure-associated deaths per 100,000 by top 3 underlying causes of death in the United States from 1999 to 2024.

| **Interval of years** | APC (95% CI) |
| --- | --- |
| **Overall** |  |
| 1999–2018 | 4.8%* (4.3 to 5.5) |
| 2018–2021 | 23.4%* (17.7 to 26.3) |
| 2021–2024 | -7.2%* (-10.9 to -3.9) |
| **Female** |  |
| 1999–2018 | 4.0%* (3.4 to 4.6) |
| 2018–2021 | 22.8%* (16.0 to 26.0) |
| 2021–2024 | -7.2%* (-10.9 to -3.9) |
| **Male** |  |
| 1999–2018 | 6.0%* (5.4 to 6.6) |
| 2018–2021 | 23.2%* (17.9 to 25.9) |
| 2021–2024 | -7.4%* (-10.6 to -4.5) |
| **NH Black or African American** |  |
| 1999–2018 | 4.2%* (3.4 to 4.9) |
| 2018–2021 | 26.2%* (18.0 to 30.2) |
| 2021–2024 | -8.4%* (-13.1 to -4.5) |
| **NH White** |  |
| 1999–2004 | 8.3%* (4.6 to 24.1) |
| 2004–2009 | 1.7% (-3.0 to 7.0) |
| 2009–2018 | 6.4%* (3.8 to 9.9) |
| 2018–2021 | 20.9%* (16.0 to 23.8) |
| 2021–2024 | -5.8%* (-9.2 to -3.2) |
| **Hispanic or Latino** |  |
| 1999–2018 | 4.5%* (3.6 to 5.4) |
| 2018–2021 | 28.1%* (20.8 to 32.3) |
| 2021–2024 | -15.4%* (-18.8 to -11.7) |
| **Northeast** |  |
| 1999–2018 | 4.8%* (4.1 to 5.4) |
| 2018–2021 | 21.0%* (14.1 to 24.5) |
| 2021–2024 | -6.3%* (-10.8 to -2.8) |
| **Midwest** |  |
| 1999–2010 | 4.0% (-1.9 to 5.5) |
| 2010–2018 | 7.1%* (4.9 to 10.0) |
| 2018–2021 | 18.1%* (12.6 to 21.1) |
| 2021–2024 | -7.2%* (-10.6 to -4.3) |
| **South** |  |
| 1999–2018 | 4.9%* (4.3 to 5.6) |
| 2018–2021 | 27.2%* (21.1 to 30.7) |
| 2021–2024 | -7.0%* (-10.5 to -3.8) |
| **West** |  |
| 1999–2018 | 4.1%* (3.4 to 4.8) |
| 2018–2021 | 20.8%* (13.1 to 24.3) |
| 2021–2024 | -8.5%* (-13.0 to -4.9) |
| **Metropolitan** |  |
| 1999–2004 | 7.3%* (5.8 to 10.5) |
| 2004-2010 | 2.2% (-0.8 to 3.3) |
| 2010-2019 | 6.6%* (6.1 to 7.3) |
| **Non-Metropolitan** |  |
| 1999–2003 | 8.7%* (5.6 to 16.9) |
| 2003-2013 | 3.8%* (0.3 to 4.6) |
| 2013-2019 | 8.2%* (6.8 to 11.1) |
| **Young Adults** |  |
| 1999–2018 | 3.6%* (2.9 to 4.3) |
| 2018–2021 | 26.0%* (17.6 to 30.2) |
| 2021–2024 | -10.0%* (-14.6 to -6.0) |
| **Middle-Aged Adults** |  |
| 1999–2018 | 4.0%* (3.0 to 4.9) |
| 2018–2021 | 26.0%* (17.6 to 30.2) |
| 2021–2024 | -10.0%* (-14.6 to -6.0) |
| **Old Adults** |  |
| 1999–2018 | 5.6%* (5.1 to 6.1) |
| 2018–2021 | 21.9%* (17.3 to 24.4) |
| 2021–2024 | -6.1%* (-8.7 to -3.5) |

**Supplemental Table 10.** Summary APCs of obesity and heart failure-related AAMR per 100,000 in the United States from 1999 to 2024.
